# The Impact of Solvent Selection: Strategies to Guide the Manufacturing of Liposomes Using Microfluidics

**DOI:** 10.3390/pharmaceutics11120653

**Published:** 2019-12-04

**Authors:** Cameron Webb, Swapnil Khadke, Signe Tandrup Schmidt, Carla B. Roces, Neil Forbes, Gillian Berrie, Yvonne Perrie

**Affiliations:** 1 Strathclyde Institute of Pharmacy and Biomedical Sciences, University of Strathclyde, 161 Cathedral Street, Glasgow G4 0RE, UK; cameron.webb@strath.ac.uk (C.W.); swapnil.khadke@strath.ac.uk (S.K.); signe.schmidt@strath.ac.uk (S.T.S.); carla.roces-rodriguez@strath.ac.uk (C.B.R.); neil.forbes@strath.ac.uk (N.F.); gillian.berrie@strath.ac.uk (G.B.); 2 Department of Infectious Disease Immunology, Center for Vaccine Research, Statens Serum Institut, Artillerivej 5, 2300 Copenhagen S, Denmark

**Keywords:** liposomes, microfluidics, solvents, formulation, particle size, pegylation, alcohol

## Abstract

The aim of this work was to assess the impact of solvent selection on the microfluidic production of liposomes. To achieve this, liposomes were manufactured using small-scale and bench-scale microfluidics systems using three aqueous miscible solvents (methanol, ethanol or isopropanol, alone or in combination). Liposomes composed of different lipid compositions were manufactured using these different solvents and characterised to investigate the influence of solvents on liposome attributes. Our studies demonstrate that solvent selection is a key consideration during the microfluidics manufacturing process, not only when considering lipid solubility but also with regard to the resultant liposome critical quality attributes. In general, reducing the polarity of the solvent (from methanol to isopropanol) increased the liposome particle size without impacting liposome short-term stability or release characteristics. Furthermore, solvent combinations such as methanol/isopropanol mixtures can be used to modify solvent polarity and the resultant liposome particle size. However, the impact of solvent choice on the liposome product is also influenced by the liposome formulation; liposomes containing charged lipids tended to show more sensitivity to solvent selection and formulations containing increased concentrations of cholesterol or pegylated-lipids were less influenced by the choice of solvent. Indeed, incorporation of 14 wt% or more of pegylated-lipid was shown to negate the impact of solvent selection.

## 1. Introduction

Liposomes are a versatile group of nanoparticles, which are approved for use in the clinic to improve the delivery of cancer agents, antifungal agents and vaccine adjuvants [1,2]. Currently, most approved liposome products contain already-approved drugs (e.g., amphotericin B, doxorubicin, bupivacaine, verteporfin) [3]. Recently, there has been a rise in follow-on ‘nanosimilars’ or generic liposome products being approved for use. Indeed, this has resulted in new recommendations for the naming of liposomal medicines such that the qualifier ‘liposomal’ or ‘pegylated liposomal’ should be added to the name. This recommendation, made jointly by the EMA’s human medicines committee and the Coordination Group for the mutual Recognition and Decentralised Procedures, aims to provide clearer distinctions between liposomal and non-liposomal formulations and hence avoid medication errors [4].

However, despite the increase in their use, the manufacturing processes used in the production of liposomes remains relatively unchanged, with time-consuming, multi-stage batch production procedures being common. Furthermore, in many liposome research studies, small-scale methods are still used that do not offer realistic line-of-sight to a manufacturing process. This reduces our ability to translate liposome technology from bench to patient. To address this, new manufacturing methods are being investigated, which can offer scale-independent production. In particular, microfluidics is being widely investigated as a scale-independent production method (e.g., [5,6,7]). This method generally relies on the bottom up self-assembly of the liposomes by controlled mixing of lipids dissolved in an aqueous soluble organic solvent with an aqueous buffer [8,9]. Using this method, we have demonstrated the ability to entrap small molecules [10,11], nucleic acids [12] and proteins [13,14] within liposomes. We have also demonstrated the scale-independent production of liposomal adjuvants using this method [15,16,17,18].

**Figure 1:**
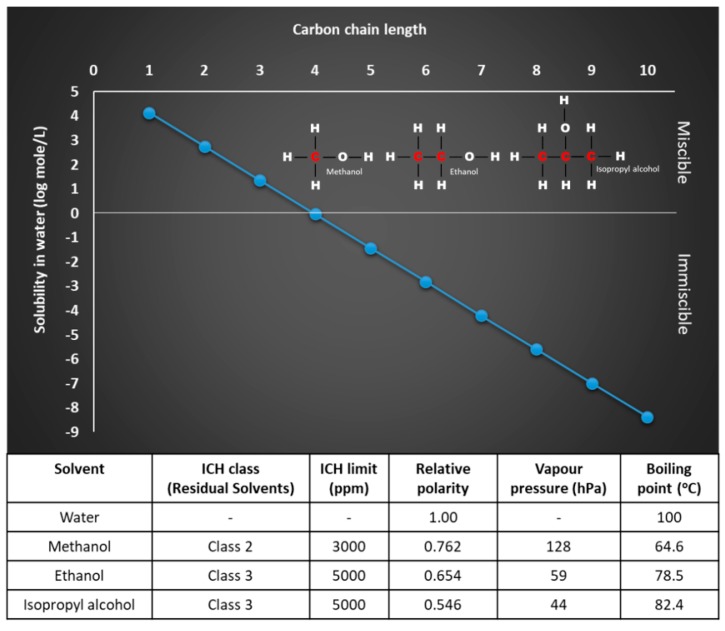
The relationship between aqueous solubility and the number of carbon atoms in alcohols determined by surface tension (adapted from [19]) and physical properties of methanol, ethanol, and isopropanol.

When adopting microfluidics as a production method for liposomes, the lipids are dissolved in an organic solvent (normally alcohol), which is then mixed with the aqueous phase to promote nanoprecipitation and liposome production. Therefore, the solvent selected must dissolve the lipid component and be water soluble. The water miscibility of organic solvents is directly related to carbon chain length and surface tension as shown in Figure 1 (adapted from [19]). As the length of the carbon chain increases, the polar OH group becomes a smaller component of the solvent molecule. The solubility and the polarity of alcohol decreases correspondingly. The solubility of organic solvents in water is also driven by hydrogen-bonding interactions between water and solvent. For example, water and short-chain solvents such as ethanol are completely miscible due to the ability of the ethanol molecules to form hydrogen bonds with water molecules as well as with each other [20]. However, when considering solvent selection for use in microfluidics, lipid solubility and water miscibility are not the only factors. Whilst in the purification process solvent is removed, working with solvents that have low toxicity potential and defined as Class 3 in the ICH Q3C (R6) [21] (e.g., ethanol and isopropanol (IPA)) is preferable followed by those in Class 2 (e.g., methanol) (Figure 1).

**Figure 2:**
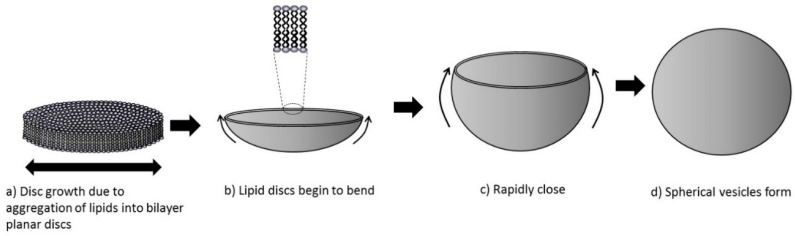
A hypothesised liposome formation mechanism (adapted from Zook and Vreeland [26]). The process starts with the aggregation of lipids in discs (**a**). It is proposed that the hydrophobic chains around the edges are stabilised by alcohol molecules. As the alcohol concentration reduces these lipid discs bend (**b**), rapidly close (**c**), and form liposomes (**d**).

Typically, IPA has been used as the lipid solvent in microfluidic processes [8,22,23,24], with fewer studies exploring other solvents such as ethanol as a less toxic alternative for medicinal applications, which would also comply with routine industrial processes [25]. However, the choice of solvent used during microfluidics may have an impact; Zook and Vreeland hypothesised that liposomes formed due to the alcohol and aqueous phase mixing, increasing the polarity of the solvent, which caused the lipids to become progressively less soluble and self-assemble into planar lipid bilayers [26]. The authors noted that as the planar bilayers increase in size, to reduce the surface area of hydrophobic chains exposed to the polar solvent around the edge of the discs, they bend and form spherical liposomes (Figure 2) [12]. Given that this change in polarity will be dependent on the initial polarity of the alcohol selected, solvent polarity is a critical material attribute to consider as it will impact both on initial lipid solubility but also on the nanoprecipitation process. Therefore, the aim of this work was to evaluate the impact of solvent selection on the formulation of liposomes in terms of their physicochemical parameters, such as particle size, polydispersity index, liposome stability and drug/protein loading. To achieve this, we initially employed a low volume high throughput microfluidic system as a screening process, followed by more detailed formulation studies using larger volume microfluidic systems. 

## 2. Materials and Methods 

### 2.1. Materials

Soy bean phosphatidylcholine (SoyPC), hydrogenated soy phosphatidylcholine (HSPC), 1,2-distearoyl-*sn*-glycero-3-phosphocholine (DSPC), 1,2-distearoyl-*sn*-glycero-3-phosphoethanolamine-*N*-[methoxy(polyethylene glycol)-2000] (DSPE-PEG2k) were obtained from Lipoid (Ludwigshafen, Germany). 1,2-dimyristoyl-*sn*-glycero-3-phospho-(1′-rac-glycerol) (DMPG), 1,2-dioleoyl-*sn*-glycero-3-phosphoethanolamine (DOPE), 1,2-dimyristoyl-*sn*-glycero-3-phosphocholine (DMPC), 1-palmitoyl-2-oleoyl-glycero-3-phosphocholine (POPC), 1,2-dioleoyl-3-trimethylammonium-propane (chloride salt) (DOTAP) were purchased from Avanti polar lipids, Alabaster, AL, USA. Cholesterol (Chol), chicken egg ovalbumin (OVA), and propofol were acquired from Sigma-Aldrich (St. Louis, MO, USA). Phosphate buffered saline was acquired from Oxoid Ltd., Basingstoke, UK. Tris-base was obtained from IDN Biomedical Inc. (Aurora, OH, USA). Methanol (MeOH), ethanol (EtOH) and isopropanol (IPA) were obtained from Fisher Scientific, Loughborough, UK. All solvents and other chemicals were used at analytical grade, and mQ-water was provided by an in-house system.

### 2.2. Microfluidic Production of Liposomes

#### 2.2.1. Initial Rapid Small-Scale Screening Studies

The liposomes were prepared with a phospholipid:cholesterol ratio of 3:1 (SoyPC and POPC) or 2:1 (HSPC, DMPC and DSPC) weight ratio. Pegylated liposomes (DSPC:Chol:DSPE-PEG2k) were prepared at a 2:1:1 *w*/*w*. Anionic liposomes were prepared from DMPC:Chol:DMPG or DSPC:Chol:DMPG at 10:5:4 *w*/*w* and cationic liposomes were prepared from DOPE:DOTAP at 1:1 *w*/*w*, HSPC:Chol:DOTAP at 1:2:4 *w*/*w* and HSPC:Chol:DOTAP:DSPE-PEG2k at 1:2:4:0.5 *w*/*w*. These formulations were selected to represent commonly reported formulations. For microfluidic production, the lipids were dissolved in methanol, ethanol or isopropanol at 4 mg/mL. For initial rapid screening, the formulations were prepared using a Spark^TM^ (Precision Nanosystems Inc., Vancouver, Canada). Disposable microfluidic chips were loaded with 31 µL lipid stock and 93 µL PBS or Tris buffer in the reaction chambers, respectively. The receiving chamber was filled with 124 µL PBS or Tris buffer (pH 7.4, 10 mM). With formulations prepared with OVA, the protein was added to the PBS at a concentration of 1 mg/mL, whilst for formulations with propofol, the drug was added to the lipid stock solution at 1 mg/mL. The formulations were processed at setting 8–10, and the product transferred to a glass vial and further diluted with 1000 µL PBS or deionised water. All formulations were prepared at room temperature.

#### 2.2.2. Preparation of Liposomes Using a Bench-Scale System

Liposomes were prepared with DSPC:Chol (2:1 to 8:1 *w*/*w* ratio; 11 to 33% cholesterol content) and DOPE:DOTAP (1:1 *w*/*w* ratio) and 4 mg/mL initial lipid concentration by using a Nanoassemblr^TM^ (Precision Nanosystems Inc.). Increasing levels of DSPE-PEG2k (0 to 25 wt%) were also tested with the DSPC:Chol formulation. A range of alcohol mixtures were tested as the organic phases: methanol/ethanol, methanol/IPA and ethanol/IPA at combinations of 100/0, 75/25, 50/50, 25/75 and 0/100 *v*/*v*%. Tris-buffer (10 mM, pH 7.4) was used as the aqueous phase at a flow rate ratio of 1:1 for the DOPE:DOTAP. For DSPC:Chol formulations, PBS at a flow rate ratio of 3:1 was used. Again, all formulations were prepared at room temperature.

### 2.3. Characterization of Particle Size, Polydispersity and Zeta Potential by Using Dynamic Light Scattering

The particle sizes, measured as the hydrodynamic diameters, polydispersity indexes (PDI) and zeta potentials were measured by dynamic light scattering using a Zetasizer Nano ZS (Malvern Instruments Ltd., Worcestershire, UK) equipped with a 633 nm laser and a detection angle of 173°. The samples were measured and the values of water were used for refractive indexes and viscosity. Zetasizer Software v.7.11 (Malvern Instruments Ltd.) was used for the acquisition of data.

### 2.4. Removal of Free Drug with Tangential Flow Filtration (TFF)

The OVA and propofol in the drug loaded samples were removed by tangential flow filtration by using a Krosflo KR2i TFF system (Waltham, MA, USA) equipped with an mPES 300 kD or 750 kD column. The sample volume was 1 mL and the purification process was repeated 12 times. All samples were recovered at the initial volume.

### 2.5. Characterisation of Drug Loading

OVA (43 kDa) and propofol (178 Da) were selected as a water soluble and bilayer soluble drug respectively to consider drug loading. These were selected as we previously investigated drug loading via microfluidics using these two moieties [11,13]. The content of propofol in propofol-loaded formulations after purification with TFF was determined using HPLC as described in [27]. Propofol loading was calculated as the percent of propofol in the liposome formulation after TFF purification to the initial concentration in the untreated sample. The content of OVA in the OVA loaded samples after purification with TFF was quantified using either the bicinchoninic acid assay (BCA) (Thermo Fisher Scientific, Waltham, MA, USA) or via HPLC using a modified published method [28]. A Jupiter 5 µm C5 300A column 4.6 mm i.d. × 250 mm length (Phenomenex, Macclesfield, UK) was used with a gradient flow (0.1% TFA in water (A), 0.1% TFA in acetonitrile (B)). A UV detector was fitted at 210 nm for all OVA loaded samples (retention time 10.6–14 min) whilst 280 nm was used for the protein release study (retention time 8–14 min). 

### 2.6. Morphological Characterisation of Liposomes via CryoTEM

Samples were prepared by placing 5 µL of liposomes onto a 400-mesh lacey carbon-coated grid using single sided blotting for 2 s then immediately immersing the sample grid into nitrogen cooled ethane (100% ethane). The liposome morphology was then observed using the Joel Jem F-200 microscope (Joel, Tokyo, Japan) at liquid nitrogen temperature and 200 kV.

### 2.7. Liposome Stability Studies

Neutral liposomes (DSPC:Chol 2:1 *w*/*w*) were produced using microfluidics at a 3:1 FRR and 15 mL/min TFR (4 mg/mL initial lipid concentration) in either MeOH, EtOH or IPA. Solvent was removed by TFF (12 mL wash cycle per mL of sample). Liposome suspensions were then stored at 2–8 °C in the fridge and their size and PDI was measured over 7 days with the Zetasizer Nano ZS (Malvern Instruments Ltd., Worcestershire, UK) using a 1/10 dilution with purified water.

### 2.8. Drug Release Studies

Ovalbumin loaded entrapped within DSPC:Chol liposomes were produced using microfluidics at a 3:1 FRR and 15 mL/min TFR (16 mg/mL initial lipid and 1 mg/mL initial Ovalbumin) using MeOH, EtOH or IPA. These formulations were then purified via TFF as previously described. 1 mL of purified formulation was added into a 300 kD float-a-lyzer™ (Spectrum™, Breda, The Netherlands) in the presence of 20 mL PBS (pH 7.4 ± 0.2). The samples were incubated at 37 °C with agitation and at 0, 24, 48 and 72 h 100 µL of the sample was removed and protein retention was quantified using the described RP-HPLC method.

## 3. Results

### 3.1. Rapid Pre-Screening of Liposome Manufacture Indicates that Liposome Size Can Be Influenced by Solvent Selection

**Figure 3:**
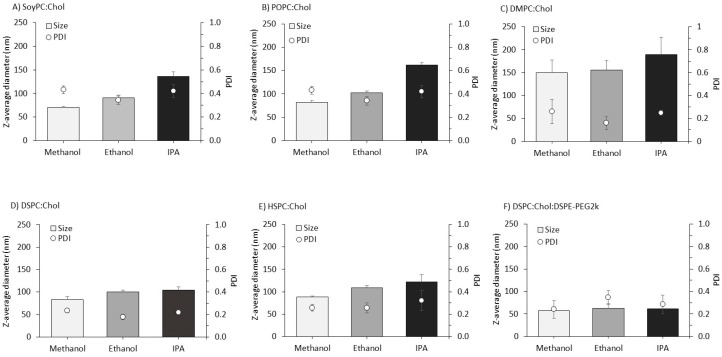
Initial low-volume, high-throughput screening of liposomes formulated using different solvents during microfluidic production. Liposomes were prepared using a low volume, high throughput screening system (Spark^TM^). Different water miscible organic solvents (methanol, ethanol or IPA) were used in combination with the aqueous phase. A flow rate ratio of 3:1 aqueous:organic phase was used with 4 mg/mL total lipid concentration in the organic phase. Various liposome formulations were tested: (**A**) soyPC:Chol 3:1 *w*/*w*, (**B**) POPC:Chol 3:1 *w*/*w*, (**C**) DMPC:Chol 2:1 *w*/*w*, (**D**) DSPC:Chol 2:1 *w*/*w*, (**E**) HSPC:Chol 2:1 *w*/*w*, (**F**) DSPC:Chol:DSPE-PEG2k 2:1:1 *w*/*w*. Columns represent particle size and open circles represent PDI. Results represent mean ± SD from three independent batches.

To investigate the impact of solvent selection during microfluidic production on the properties of liposomes, we initially screened a panel of liposome formulations using a small-volume high-throughput microfluidic system (Spark^TM^; Precision Nanosystems Inc.). Initially a panel of six liposome formulations were tested, which contained a combination of a phosphatidylcholine and cholesterol, and in one formulation we also incorporated a PEG-coating (Figure 3). The results showed that the solvent selected could have an impact on liposome size depending on the formulation. In general, the SoyPC and POPC liposome formulations (PC:Chol 3:1 *w*/*w*) tended to show a greater impact from the solvent adopted in terms of particle size. For example, with both formulations the liposome particle size approximately doubled when liposomes were formulated using IPA compared to methanol. With the SoyPC:Chol formulation, liposomes increased in size from 70 to 135 nm as we moved from methanol to IPA (Figure 3A) and the POPC:Chol liposomes increased from 81 to 161 nm (Figure 3B). When the liposomes were prepared from DMPC, DSPC and HSPC (2:1 *w*/*w* with cholesterol; Figure 3C, D and E respectively), there was less impact on particle size as we decreased the solvent polarity. For example, DMPC:Chol liposomes increased in size from 149 to 190 nm as we switched from methanol to IPA (Figure 3C), DSPC:Chol increased from 83 to 104 nm (Figure 3D) and HSPC:Chol liposomes increased from 88 to 122 nm (Figure 3E). Across all these formulations, the general trend of increasing liposome size in terms of IPA > ethanol > methanol could be seen with the exception of the pegylated formulation. When DSPE-PEG2k was included in the formulation (DSPC:Chol:PEG2k) the liposomes were similar in size (approximately 60 nm) irrespective of the solvent used in their manufacture (Figure 3F). Across all formulations, the PDI was in the range of 0.2 to 0.4, with no impact from the solvent used (Figure 3). 

**Figure 4:**
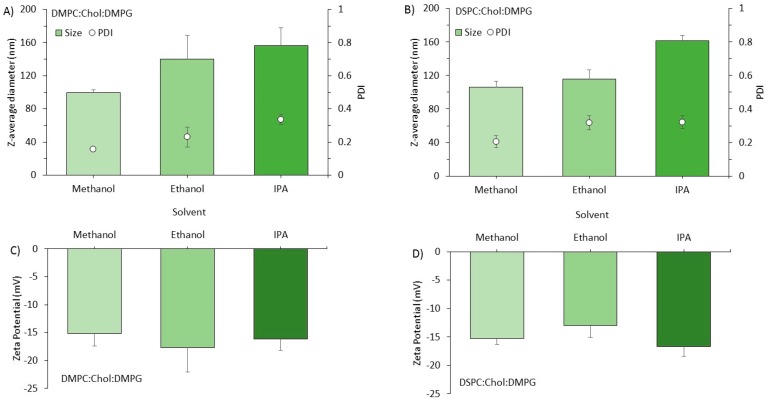
The effect of solvent selection on the particle size of anionic liposomes. Liposomes were prepared using different water miscible organic solvents (methanol, ethanol or IPA) and PBS as aqueous phase on the Spark^TM^ microfluidic system as shown in Figure 3. A flow rate ratio of 3:1 aqueous:organic phase was used with 4 mg/mL total lipid concentration in the organic phase. Particle size and PDI (columns and open circles, respectively) are shown for (**A**) DMPC:Chol:DMPG 10:5:4 *w*/*w*, and (**B**) DSPC:Chol:DMPG 10:5:4 *w*/*w* formulations. The zeta potential for (**C**) DMPC:Chol:DMPG 10:5:4 *w*/*w*, and (**D**) DSPC:Chol:DMPG 10:5:4 *w*/*w* is also shown. Results represent mean ± SD from three independent batches.

**Figure 5:**
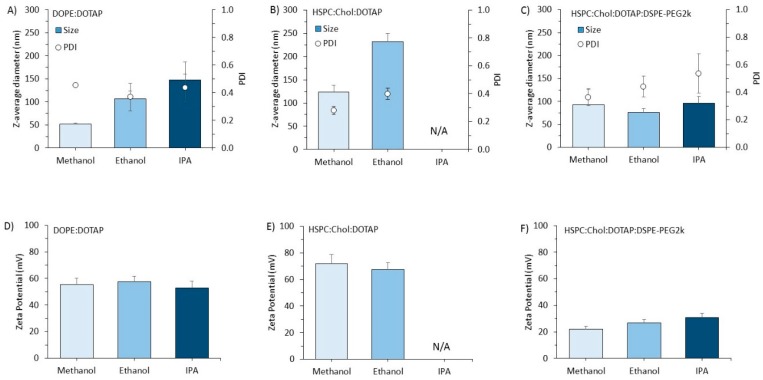
The effect of solvent selection on the particle size of cationic liposomes. Liposomes were manufactured as outlined in Figure 3. A flow rate ratio of 3:1 aqueous:organic phase was used with 4 mg/mL total lipid concentration in the organic phase and Tris buffer (10 mM, pH 7.4) as the aqueous phase. Particle size and PDI (columns and open circles, respectively) for (**A**) DOPE:DOTAP 1:1 *w*/*w*, (**B**) HSPC:Chol:DOTAP 1:2:4 *w*/*w* and (**C**) HSPC:Chol:DOTAP:DSPE-PEG2k 1:2:4:0.5 *w*/*w*. The zeta potential for these three formulations are shown in (**D**), (**E**) and (**F**) respectively. Results represent mean ± SD from three independent batches.

To further explore the impact of solvent selection, we also tested a selection of anionic (Figure 4) and cationic (Figure 5) formulations. In terms of the anionic formulations (DMPC:Chol:DMPG and DSPC:Chol:DMPG), again we saw the general trend of increasing particle size (from approximately 100 to 160 nm) for both the DMPC and DSPC formulations (Figure 4A,B respectively) as we progressed from methanol to IPA. Again, for all formulations the PDI remained in the range of 0.2 to 0.4 (Figure 4). The impact of solvent selection on the zeta potential was also tested; no significant difference was seen across the anionic liposome formulations irrespective of the solvent selected for their production (−15 to −25 mV; Figure 4C,D). 

With the cationic (DOPE:DOTAP) formulations, again we see a trend of increasing liposome size as we decrease the polarity of the solvent, with sizes increasing from approximately 50 nm, to 100 nm to 150 nm when formulated in methanol, ethanol and IPA respectively (Figure 5A). When the HSPC:Chol:DOTAP formulation was made in methanol or ethanol, particle size increased from 120 nm to 230 nm, respectively (Figure 5B). With this formulation, IPA could not be tested due to precipitation of the formulation. Interestingly, when PEG was added into this formulation, precipitation was not an issue and the solvent adopted in the microfluidic process had no impact on particle size (76–96 nm irrespective of the solvent used; Figure 5C) and the PDI remained in the range of 0.2 to 0.4. As with the anionic formulations, the zeta potential of the formulations were not influenced by the solvent selected with DOPE:DOTAP and HSPC:Chol:DOTAP formulations being in the range of 50 to 60 mV (Figure 5D,E) and the pegylated cationic formulation being 20–30 mV (Figure 5F). 

**Figure 6:**
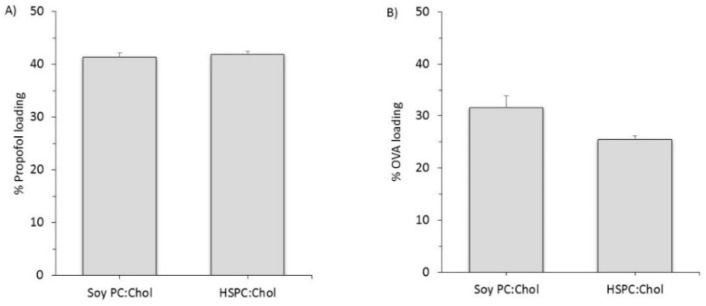
Liposome loading efficiencies for the (**A**) bilayer loaded drug (propofol, 1 mg/mL in MeOH) and (**B**) aqueous core loaded drug (OVA, 1 mg/mL in PBS) produced using rapid through-put microfluidics (Spark^TM^). Liposomes were prepared with soyPC:Chol and HSPC:Chol, 3:1 *w*/*w*, 10 mg/mL in MeOH. Results represent mean ± SD from three independent batches.

The applicability of using this low volume microfluidic system (Spark^TM^) as a high-throughput screening tool for liposome formulations was also tested in terms of drug loading. Figure 6 demonstrates that even with low volume formulation testing (down to 100–250 uL) drug loaded liposomes can be prepared rapidly for initial screening with drug loading of low soluble drugs (propofol) and large biologicals (OVA) being achieved at levels comparable (40% for propofol [13] and 25–30% for OVA [28]) to those previously reported with the larger bench-top microfluidic systems.

### 3.2. The Impact of the Solvent Is Dependent on the Liposome Formulation

**Figure 7:**
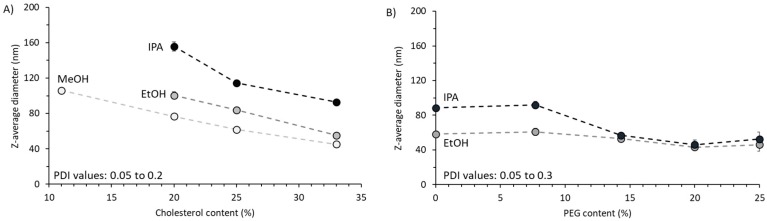
The effect of increasing cholesterol content and PEG-lipid in liposomes manufactured using microfluidics and different solvents. (**A**) Cholesterol was increased from 11 to 33 wt% in DSPC:Chol liposomes and (**B**) PEG-lipid content was increased from 0 to 25% within DSPC:Chol:PEG2k liposomes. Liposomes were prepared at a FRR of 3:1 and TFR of 15 mL/min for DSPC:Chol liposomes and 12 mL/min for DSPC:Chol:DSPE-PEG2k liposomes. Initial lipid concentration for both formulations was 4 mg/mL. Results represent mean ± SD from three independent batches.

To further consider the impact of the liposome formulation in combination with solvent selection, DSPC:Chol liposome formulations were prepared with varying cholesterol content from 11 to 33 wt% and the bench-top NanoAssemblr^TM^ was employed. As this system was designed for the formulation and development of nanomedicines, it offered the ability for liposomes to be collected during the steady state microfluidic production and hence produced homogeneous suspensions with PDI commonly below 0.2. Figure 7A shows that as we increased the cholesterol content, the liposome size reduced with all three solvents. The results also showed the trend of liposome size increased as we progressed from methanol, to ethanol, to IPA irrespective of the cholesterol content used. However, at higher cholesterol concentrations the difference in size between the liposomes formed in the different solvents was reduced (Figure 7A). Furthermore, at low cholesterol concentrations (11 wt%) liposomes could only be manufactured using methanol (Figure 7A). Attempts to manufacture this liposome formulation with ethanol or IPA resulted in large aggregates forming. 

The addition of PEG-lipid to the DSPC:Chol (2:1 *w*/*w*; 33 wt% cholesterol) was then investigated with ethanol and IPA (Figure 7B). At low PEG lipid content (8%), we saw that the choice of solvent significantly (*p* < 0.05) impacted particle size (60 vs. 90 nm for ethanol and IPA respectively; Figure 7B). However, as we increased the PEG-lipid content to 14% or more, we saw the effect of solvent was negated (Figure 7B).

### 3.3. Varying the Solvent Mixture Composition Impacts on Liposome Particle Size

**Figure 8:**
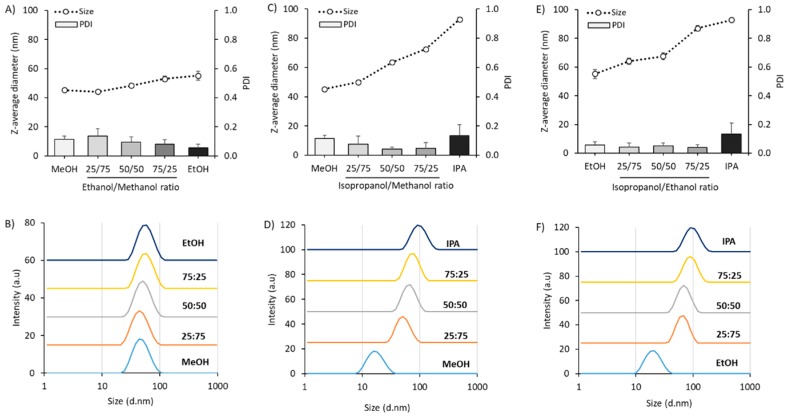
Investigating the effect of solvent mixtures on physical liposome characteristics. Particle size and PDI recorded with lipids dissolved in (**A**) Methanol:Ethanol mix 0–100%, (**C**) Methanol:Isopropanol mix 0–100%, (**E**) Ethanol:Isopropanol mix 0–100% with intensity plots for each respective solvent mix shown in (**B**), (**D**) and (**F**). Liposomes were composed of DSPC:Chol (2:1 *w*/*w*), which were produced using a FRR of 3:1; 15 mL/min TFR and initial lipid concentration of 4 mg/mL. Results represent mean ± SD from three independent batches.

To further study the link between solvent selection and particle size, Figure 8 explores in detail the impact of solvent mixtures on the particle size attributes of liposomes formulated from DSPC:Chol. From Figure 8A, we can see that as we increased the ethanol content within the methanol/ethanol solvent mix from 0 to 100%, the particle size increased only moderately from 45 to 55 nm with the PDI remaining below 0.2 and the suspensions were unimodal in nature (Figure 8B). When we consider the impact of IPA on the formulations, the increase in size was more notable. DSPC:Chol liposomes prepared using mixtures of methanol/IPA showed an increase in size from 45 nm up to 93 nm as we increased the IPA content from 0 to 100% (Figure 8C), again with the PDI remaining below 0.2 and the particle size being unimodal (Figure 8D). When these liposomes were prepared using mixtures of ethanol/IPA, we could similarly see an increase in size (from 55 to 93 nm as we go from 0 to 100% IPA; Figure 8E). Again, the PDI of all formulations remained <0.2 and were unimodal in nature (Figure 8F). These results show that we can control the liposome particle size via controlling the solvent mixture. 

**Figure 9:**
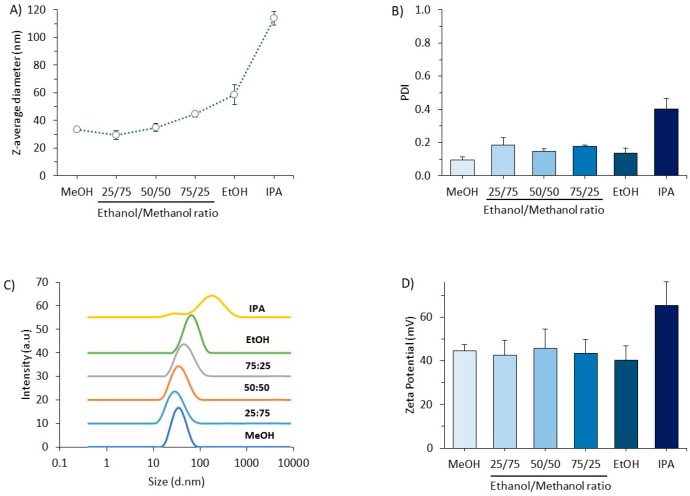
Controlling the vesicle size of cationic liposomes using solvent mixtures. DOPE:DOTAP (1:1 *w*/*w*) liposomal formulations were prepared using the NanoAssemblr^TM^ bench-scale system. A flow rate ratio of 1:1 aqueous:organic phase was used with 4 mg/mL total lipid concentration in the organic phase and Tris buffer (10 mM, pH 7.4) as aqueous phase. Solvent mixtures of methanol/ethanol and IPA were tested. Results are shown as (**A**) Particle size, (**B**) PDI, (**C**) Intensity plots and (**D**) Zeta potential of the formulations. Results represent mean ± SD from three independent batches.

To test if similar size control could be achieved with cationic formulations, again a range of methanol/ethanol solvent mixtures were tested (Figure 9). Once, again the particle size was controlled through the polarity of the solvent mixture. By increasing the ratio of ethanol, the liposome size increased from 33 to 58 nm (Figure 9A) with the PDI remaining below 0.2 and the formulations being unimodal in nature (Figure 9B,C). Again formulating these formulations with IPA increased the size (up to 120 nm; Figure 9A) but did increase the polydispersity of the formulation (Figure 9B,C). However, across all the formulations, the cationic zeta potential remained high (40–60 mV; Figure 9D).

### 3.4. Solvent Choice Influences Liposome Morphology and Initial Protein Loading but Does Not Impact Liposome Stability nor Release Attributes

**Figure 10:**
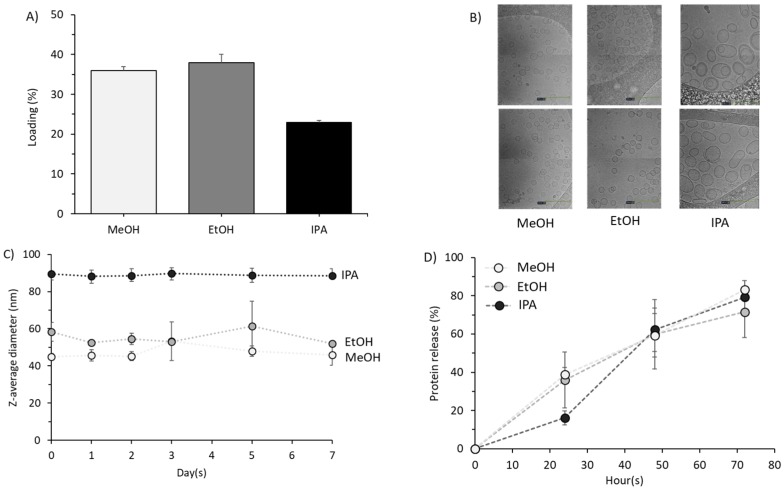
Solvent selection and its impact on OVA encapsulation, morphology, liposome stability and protein release. (**A**) Protein entrapment efficiency of liposomes loaded with 0.25 mg/mL OVA with an initial lipid concentration of 4 mg/mL produced using methanol, ethanol or isopropanol. (**B**) the morphology of liposomes obtained by cryoTEM in the respective solvents. (**C**) Stability of ‘empty’ liposomes stored at 2–8 °C over 7 days at an initial lipid concentration of 4 mg/mL. (**D**) Protein release profiles of liposomes (initial concentration of 16 mg/mL loaded with 1 mg/mL OVA) was investigated over 72 h at 37 °C to access if solvent selection impacted membrane permeability. Liposomes were composed of DSPC:Chol (2:1 *w*/*w*) and produced at a 3:1 FRR and 15 mL/min. Results represent mean ± SD from three independent batches.

To further investigate the impact of solvent choice during microfluidic manufacturing, DSPC:Chol (2:1 *w*/*w*) were prepared incorporating protein (OVA) (Figure 10). From the results in Figure 10A, we can see that there was no significant difference in protein loading for liposomes prepared with methanol and ethanol (35–40%; Figure 10A), but protein loading was significantly (*p* < 0.05) reduced when IPA (20%; Figure 10A) was used for the manufacturing process. Interestingly, when considering the morphology (Figure 10B), we can see that liposomes formed as small unilamellar vesicles when prepared using methanol or ethanol, whilst those formed with IPA were larger and had one or two bilayers. In terms of stability, all three liposome formulations showed good short-term stability when stored at 2–8 °C (Figure 10C) with no significant difference in size being noted over the length of the study. Similarly, there was no significant difference in protein release profiles of the liposomes prepared using methanol, ethanol or IPA with approximately 80% protein release after 72 h (Figure 10D).

## 4. Discussion

Microfluidic production offers new manufacturing strategies for the production of liposomes and other nanoparticles. This method can be scale-independent [16,29] and thus gives formulation scientists a direct route to translate their research off the bench into the clinic. When considering liposomes, there are a range of parameters that are critical quality attributes. Amongst these, vesicle size is particularly important given its impact on biodistribution, which is a key feature in the ability of liposomes to improve drug delivery, enhance efficacy and reduce off target toxicity. It is widely reported that critical process parameters that influence the size of nanoparticles produced via microfluidics include the micromixer design (e.g., [30]) and the rate of mixing (e.g., [13,16,17,31]). Within this study, we demonstrated that solvent selection was also a critical process parameter in the manufacture of liposomes using microfluidics as it could impact on particle size. Furthermore, we demonstrated that alcohol mixtures could be used to manipulate liposome size. However, the sensitivity of the liposome particle size to solvent selection was dependent on the given liposome formulation. This can be tested using rapid low volume screening protocols (as outlined in Figure 3, Figure 4, Figure 5 and Figure 6), which is particularly useful in early studies where lipids and active pharmaceutical ingredients may be limited. However, changes in particle size when switching between methanol and ethanol were generally low (Figure 7, Figure 8 and Figure 9). When produced by microfluidics, liposome sizes tended to increase with decreased solvent polarity across all the formulations tested and size control of the liposomes could be achieved using solvent mixtures (Figure 8 and Figure 9). Furthermore, increasing concentrations of cholesterol or pegylated-lipid formulations tended to reduce the impact of the solvent selections, and formulations containing over 14% PEG-lipid did not show a notable sensitivity to the alcohol used in their manufacture (Figure 7). Despite differences in morphology, the liposomes formulated using methanol, ethanol or IPA all showed good stability and similar protein release profiles (Figure 10).

A range of models have been proposed for the self-assembly of liposomes due to the mixing of water miscible solvents and recently Zook and Vreeland proposed a non-equilibrium model [26]. Their model proposed that liposome size was determined by two factors: 1) the growth rate of planar bilayer discs and 2) the rate the discs close into spherical vesicles (Figure 2). In their studies, Zook and Vreeland [26] tended to focus their investigations on considering the impact of the rate of disc closure, and the authors showed that the radius of the liposomes formed during their microfluidic process was proportional to the ratio of membrane bending elasticity modulus to the hydrophobic edges of the lipid discs formed. In general, the membrane elasticity modulus of a bilayer is higher with longer alkyl chain length/high transition temperature lipids (i.e., DSPC > DPPC > DMPC in terms of bending elastic modulus, and a higher elastic modulus indicates a more rigid membrane). Therefore, bilayer discs formed during the microfluidic process (as shown in Figure 2A) should be more rigid and less able to bend when the membrane is at or below the bilayer transition temperature [26]. Thus, at room temperature high transition temperature bilayer formulations would form larger liposomes. Indeed, in discussing their model, Zook and Vreeland note that temperature is a key consideration and increasing the temperature above the lipid transition temperature will increase elasticity and hence reduce particle size [26]. These findings were not in line with the results shown in Figure 3 (e.g., DMPC vs. DSPC formulations) nor with our previous findings [13]; we show that increasing the carbon chain length of the PC lipid results in smaller liposomes being formed. Similarly, we have previously shown that during the microfluidic production, liposomes produced at room temperatures or at a temperature above the main lipid transition temperature were similar in size; we were able to prepare DSPC:Chol liposomes of the same size at production temperatures from 20 to 60 °C. This demonstrates that there was no requirement to work above the lipid transition temperature during the microfluidic manufacturing process [13]. Therefore, in our studies, the impact of temperature on membrane elasticity is not a key contributing factor controlling liposome size. However, it can be useful to use elevated temperatures to improve the solubility of some lipids in solvents during the processes irrespective of their Tc (e.g., [16]).

When considering bilayer rigidity, cholesterol content is also a key factor as the transition temperature of liposome bilayers (Tc) varies when mixtures of different hydrocarbon chain length phospholipids are compared to pure lipids [32]. In the absence of cholesterol, the hydrocarbon chains of the lipids in the bilayer crystallize into the rigid-crystalline phase which results in, for example, the Tc for DSPC liposomes being 55 °C [32]. However, with the addition of 33 mol% cholesterol, this transition temperature is no longer detectable [33]. Similarly, the large head-group component of charged lipids or pegylated lipids can reduce/negate the transition temperature of lipid bilayers by inhibiting the packaging of the lipids into the rigid-crystalline phase. Moreover, it has also been shown that adding cholesterol to liposome bilayers tends to increase the elasticity of the membrane [34,35]. From Figure 7, we can see that increasing the cholesterol content within liposomes resulted in reduced vesicle sizes, and this may be attributed to the increased elasticity and more rapid closure of the discs into smaller liposomes. The presence of higher levels of cholesterol and PEG-lipid was also shown to negate the effect of solvent selection on particle size. This suggests that the geometric packaging of these lipids within a bilayer and subsequently into a liposome may overrule the impact of solvent choice.

Alcohols are known modulators of bilayer properties and this may explain why temperature and lipid bilayer transition temperature are not factors in our process. Whilst the alcohol is removed from the liposome formulations after production (via tangential flow filtration within our studies), during the microfluidic mixing phase this is a consideration. There are various studies which demonstrate the ability of alcohols to change the lipid bilayer free volume and promote bilayer disorder, and it has been reported that the bilayer-modifying potency of short-chain alcohols scales linearly with their bilayer partitioning [36]. Within a bilayer, alcohols have their –OH group in the bilayer interfacial region and their hydrophobic methyl groups in the hydrophobic core of the bilayer and thus they disrupt the bilayer packing [37]. Thus, the impact of the alcohol on the bilayer will depend both on the alcohol and the composition of the bilayer [36]. Indeed, the ability of ethanol to enhance the bilayer permeability has been exploited to improve pH gradient loading of cholesterol-free liposomes [38]. Normally, for pH gradient loading, the liposomes are incubated at temperatures above their phase transition temperature to promote drug loading. However, in cholesterol free bilayers, increased temperatures can result in the collapse of the pH gradient. Therefore, Dos Santos et al. [38] circumvented this problem by the addition of ethanol as a permeability enhancer. Ethanol is also reported to reduce the transition temperature of lipid bilayers (through promoting bilayer disorder) and at high concentrations can cause lipid interdigitation [39,40]. This disordering effect is not restricted to ethanol, and all three of the alcohols used within our studies had a bilayer disordering effect [41]. Thus, during the production of liposomes, the miscible alcohols present during the mixing process may reduce the transition temperature of the bilayer discs, hence increasing their elasticity and promoting vesicle formation. Yet, given that the release properties of the three different liposome formulations were similar (Figure 10), this suggested that on purification of the liposomes via TFF, the residual solvent was removed (as shown previously [13]) and there was no difference in fluidity/permeability of the different liposome products. 

Given that in our process the membrane elasticity and rate of membrane closure (Figure 2C,D) did not appear to be a key factor, the link between solvent selection and liposome particle size may be linked to the process outlined in Figure 2a and the initial size of the lipid discs formed [26]. During the mixing process of the alcohol and buffer, an increase in the polarity results in the formation of the discs. As we reduce the polarity of the alcohol used (going from methanol to IPA), the rate of change in polarity during the mixing process will be reduced. This may result in larger discs being formed, which subsequently close into larger liposomes. Higher concentrations of cholesterol or PEG-lipids may inhibit the packaging of lipids into larger discs. The longer chain IPA may also provide additional stabilization for the lipid discs formed, which could also contribute to the formation of larger liposomes, as suggested by Zook and Vreeland [26]. However, within their studies only IPA was used and therefore the impact of solvent selection on this stabilizing effect was not clear.

## 5. Conclusions

From our studies, we demonstrate that solvent selection is a key consideration in developing a microfluidic manufacturing process for liposome production. In terms of solvent selection, ethanol offers a range of advantages including its suitability for large scale manufacture and its Class 3 status. However, a range of solvents can be considered (e.g., octanol [42], IPA [8], methanol [30]) and solvent selection can be used to control liposome size. Furthermore, upon appropriate dilution/solvent removal the efficacy of the produced liposomes can be equivalent (e.g., [43]). Within our current studies, we outline a rapid high-throughput method for initially screening the impact of solvent selection on liposome attributes. We also show that alcohol mixtures can be used to fine-tune the liposome size. We have previously shown that buffer concentration can be used to offer size control [18]. Therefore, liposome size is under the influence of alcohol and the salt concentration of the aqueous buffer and both are key process parameters that should be considered in the development of microfluidic manufacturing processes. 

## Data Access

All data underpinning this publication is openly available from the University of Strathclyde KnowledgeBase at https://doi.org/10.15129/81b22c47-c133-41e9-86e5-8f8a45a4d443.
